# The ripple effect: How leader workplace anxiety shape follower job performance

**DOI:** 10.3389/fpsyg.2022.965365

**Published:** 2022-10-20

**Authors:** Shanshan Zhang, Lifan Chen, Lihua Zhang, Aaron McCune Stein

**Affiliations:** ^1^School of Labor and Human Resources, Renmin University of China, Beijing, China; ^2^Ipsos, New York, NY, United States

**Keywords:** workplace anxiety, cognitive interference, emotional exhaustion, epistemic motivation, job performance

## Abstract

Although the dominant view in the literature suggests that work-related anxiety experienced by employees affects their behavior and performance, little research has focused on how and when leaders’ workplace anxiety affects their followers’ job performance. Drawing from Emotions as Social Information (EASI) theory, we propose dual mechanisms of cognitive interference and emotional exhaustion to explain the relationship between leader workplace anxiety and subordinate job performance. Specifically, cognitive interference is the mechanism that best explains the link between leader workplace anxiety and follower task performance, while emotional exhaustion is the mechanism that best explains the link between leader workplace anxiety and follower contextual performance. Additionally, we examine how follower epistemic motivation serves as a boundary condition for the effect of leader anxiety on follower performance outcomes. Results from a 2-wave study of 228 leader-follower dyads in a high-tech company mostly supported our theoretical model. We conclude the study with a discussion of the theoretical and practical implications of our findings.

## Introduction

In the current “age of anxiety,” the dual influences of unfavorable environmental factors and individual psychological characteristics cause many people to experience varying degrees of anxiety in the workplace (Cheng and McCarthy, 2018). Workplace anxiety refers to employees’ reaction to the stressors generated at work. When they are anxious, employees may feel nervous, stressed, and uneasy about work-related performance (McCarthy et al., 2016; Cheng and McCarthy, 2018). Workplace anxiety has potentially detrimental consequences for both employees and their organizations. Specifically, employees who experience anxiety at work may have reduced job performance (Ford et al., 2011; McCarthy et al., 2016) and job satisfaction (Boyd et al., 2009). In addition, anxiety may prompt counterproductive behaviors (Rodell and Judge, 2009), workplace cheating behavior (Hillebrandt and Barclay, 2022), and self-interested unethical behavior (Kouchaki and Desai, 2015). From the team’s perspective, job-related anxiety may have an inverted U-shaped curvilinear relationship with team innovation (Mao et al., 2021).

Considering the harmful consequences of workplace anxiety on employee attitudes and behaviors, it is not surprising that a large number of articles have focused on the negative effects of workplace anxiety (Eysenck et al., 2007; McCarthy et al., 2016; Calderwood et al., 2018; Lin et al., 2020; Liu et al., 2020). Despite these efforts, research on the relationship between workplace anxiety and overall job performance may still be considered relatively simplistic and incomplete (Cheng and McCarthy, 2018). Previous studies mostly focused on how employees’ job-related anxiety affects their work outcomes, while ignoring the role of leader anxiety in this process. Anxiety is an emotional system of defensive behavior triggered by cues of an impending non-specific threat (Cisler et al., 2010). In leader-subordinate interactions, leader emotions have a great influence on subordinates’ behavior, emotions, and thoughts (George, 2000). More specifically, the emotions displayed by a leader will send signals to subordinates about the leader’s psychological state and possible future behavior. In turn, subordinates will likely change their behavior in response to these signals. Thus, it is essential to understand how leaders’ emotional displays affect subordinates’ job performance. Existing research on this topic has focused on two aspects. On the one hand, researchers examined positive emotions such as happiness in the rating of leaders’ negotiation latitude (Newcombe and Ashkanasy, 2002), leaders’ effectiveness (Gaddis et al., 2004), and employee performance (Gaddis et al., 2004). On the other hand, studies on negative leader emotions have mostly focused on the influence of leader anger on employee attitudes and behaviors (Schwarzmüller et al., 2018). Anxiety, which is an important negative emotion in today’s workplace, has not received sufficient attention and research. Therefore, it is crucial to understand how leader anxiety affects subordinate performance and, in turn, the performance of their organization.

Second, prior anxiety research has focused almost exclusively on the prediction of in-role behaviors (task performance) that reflect formal job expectations. However, the effect of anxiety on extra-role behaviors beyond the job description (contextual performance) has been overlooked (Calderwood et al., 2018). Task performance is defined as the effective work output achieved by employees through the application of their skills or abilities, and is an important measure of job effectiveness (Motowidlo et al., 1997, p. 72). Contextual performance refers to behavior that is not specified in the job description but which does contribute to the organization’s positive function and performance, such as voluntarily helping coworkers, cooperating with superiors, or providing suggestions to improve the workplace (Motowidlo and Van Scotter, 1994; Van Scotter et al., 2000). Since task performance and contextual performance are different categories of behavior, they are likely to have different antecedent factors (Motowidlo and Van Scotter, 1994; Borman and Motowidlo, 1997). Therefore, it is necessary to explore the differential effects of leader anxiety on employee task and contextual performance.

**FIGURE 1:**
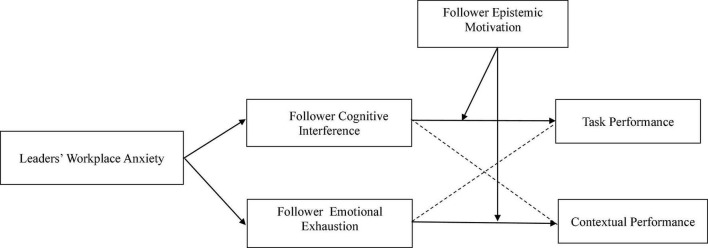
The theoretical model of our research.

Accordingly, our study aims to better understand how and when leader workplace anxiety influences subordinates’ task and contextual performance. We draw on the Emotions as Social Information (EASI) model (Van Kleef, 2009) to examine how subordinates’ cognitive interference and emotional exhaustion transmit the effect of leader anxiety on subordinate performance. Specifically, we suggest that leader anxiety can affect employee job performance by simultaneously triggering employees’ inferential processes and emotional reactions. We propose that the above mentioned inferential process occurs when workplace anxiety provides employees with informational cues that lead them to initiate deep cognitive processing to infer the social intentions behind the leader’s emotions, which can increase those employees’ cognitive interference. When employees experience cognitive interference, their task performance will suffer. We also propose that employees will have an emotional response where their leader’s negative anxiety will lead to their emotional exhaustion through the process of emotional contagion. This emotional exhaustion will reduce employees’ contextual performance. We further propose that cognitive interference better explains how leader anxiety influences employee task performance, while emotional exhaustion better explains how leader anxiety influences employee contextual performance. To provide more situational context to our research, we also examine follower epistemic motivation as a potential moderator of the relationship between leader anxiety and subordinate job performance. Epistemic motivation originates from individuals’ need for cognitive closure and refers to the motivation to engage in deep thinking (Scholten et al., 2007) and independently develop and hold well-informed conclusions about the world (Amit and Sagiv, 2013). Consequently, epistemic motivation might influence employees’ ability to process information (Mayseless and Kruglanski, 1987), including their emotional and cognitive responses to their leader’s anxiety (Van Kleef et al., 2009). Thus, we examine epistemic motivation as a moderator of the relationship between cognitive interference and emotional exhaustion and employee performance outcomes. Our theoretical model is shown in Figure 1.

Our research makes three main contributions to the literature. First, we advance the anxiety literature by deriving a conceptual framework from past theory and research to describe the mechanisms by which leader workplace anxiety affects follower performance. Based on the Emotion-as-Social-Information theory, we tested how follower emotional exhaustion and cognitive interference mediate the relationship between leader anxiety and employee job outcomes. Second, we extend the anxiety literature by simultaneously considering the influence of workplace anxiety on subordinates’ task performance and contextual performance. Because previous studies have found that task and contextual performance involve different behavior patterns and have unique antecedents (Motowidlo and Van Scotter, 1994), considering the different mechanisms through which leader anxiety affects these two aspects of performance can help organizations to implement more targeted initiatives for improving employee performance. Finally, we also contribute to the literature on the EASI model by examining the effects of a discrete emotion experienced in the workplace (anxiety) on employee behavior. Previous studies using this model have examined the effects of discrete emotions, such as anger (Van Kleef et al., 2010), disappointment (Van Kleef et al., 2006), and happiness (Van Kleef et al., 2004), on individual behavior. However, anxiety as a pervasive workplace problem has only rarely been examined.

## Theoretical development and hypotheses

### Workplace anxiety

Anxiety is defined as the tendency to experience tension and worry with regard to the appraisal of threatening situations (Spielberger, 1985; Lazarus, 1991). Spielberger’s (1985) definition highlighted the multifaceted nature of anxiety and distinguished between trait and state anxiety. Unlike general trait anxiety, which refers to an individual’s stable and general experience of anxiety, workplace anxiety is a domain-specific construct that is affected by both individual differences and workplace characteristics (Spielberger, 1966, 2013; Motowidlo et al., 1986). Workplace anxiety is defined as a negative emotional state related to work and is characterized by subjective feelings of tension and apprehension for completing job tasks (Sarason et al., 1990; Muschalla and Linden, 2012; Cheng and McCarthy, 2018). Workplace anxiety encompasses apprehension about performing specific tasks, such as job interview anxiety (Mccarthy and Goffin, 2004) or project anxiety (Wang et al., 2021). In addition, workplace anxiety is different from the related construct of neuroticism. While neuroticism reflects a tendency to experience a wide range of negative emotions such as fear and guilt, as well as emotional instability (Goldberg, 1990), workplace anxiety is a response to work-related stressors which manifests in the form of stress symptoms (Cheng and McCarthy, 2018).

In this study, we focus on leader workplace anxiety for several reasons. First, leaders not only need to complete their work tasks but also undertake important aspects of people management such as developing the relationship with subordinates or even handling complex relationships between multiple members of the organization at different levels of the hierarchy (Turner and Müller, 2005). Second, leaders act as powerful agents within the organization and their emotions have a considerable influence on the effectiveness of subordinates’ work (Van Kleef et al., 2009).

### Cognitive interference and emotional exhaustion as dual mediating mechanisms

There has been a growing body of research on workplace anxiety over the past two decades. Most of these studies have focused on the effects of anxiety on employees’ behavior. However, emotions are not simply a reflection of a person’s internal emotional state—they can also serve as social information cues that convey important information to that person. The Emotion as Social Information (EASI) theory provides a comprehensive framework to understand how emotions are interpreted and used by those who perceive them (Van Kleef, 2014). The EASI model (Van Kleef, 2009) posits that emotions can affect the perceiver’s behaviors *via* two distinct routes: inferential processes and emotional reactions. Inferential processes refer to an emotional observer’s inference about the emotion expressor’s true emotions and intentions (Beal et al., 2005). These processes require the observer to make conscious inferences about the emotions of the expresser. Individual cognitive processes and behaviors are adjusted based on information provided by other people’s emotions (Sarason et al., 1995). In this way, leader anxiety will engage subordinates’ cognitive processes, resulting in cognitive interference for the subordinate.

Emotional reactions are the processes through which emotions expressed by one individual influence the emotions of another individual who observes them (Wang et al., 2017). Unlike inferential processes, emotional reactions mean that emotional expression directly stimulates the observer’s emotional state, thus producing an emotional “interpersonal effect” (Van Kleef et al., 2010). Specifically, this process can be divided into two stages. In the first stage, emotional expressors directly spread their emotions to observers through emotional contagion (Van Kleef, 2009), which is a tendency to unintentionally and automatically capture other people’s emotions through facial expressions, language, posture, and body movements (Van Kleef et al., 2010). In the second stage, this emotion can “reappear” in the observer and influence his decision-making. This experience requires relatively little cognitive processing. In this way, followers (observers) may exhibit emotional exhaustion as a response to their leaders’ anxiety.

### The inferential processes pathway—Cognitive interference

Drawing on the EASI model, the emotional expressions of others can elicit the cognitive processes of observers, which may subsequently affect the observers’ behavior (Van Kleef, 2014; Van Kleef and Côté, 2018). As emotions have such distinct appraisal patterns, organizational members can extract useful information from each other’s emotional expressions through the cognitive process, which guides their behaviors and attitudes (Hareli and Hess, 2010). For example, when an employee is confronted with an expression of anger from her team leader, she may infer that her leader is not satisfied with the team’s current work outcomes, and decide to make an extra effort to improve the team’s performance (Van Kleef et al., 2009). Since cognitive interference mainly reflects an individual losing focus on task-related thoughts and behaviors (Sarason et al., 1986a,^2014^), we used cognitive interference as the main factor explaining the negative relationship between leader workplace anxiety and subordinate job performance. In this study, we discuss the impact of leader workplace anxiety on subordinate task performance and contextual performance separately. Borman and Motowidlo (1997) argued that when conceiving of and measuring employee job performance, it is valuable to distinguish between contextual performance and task performance.

We expect that leader workplace anxiety engages followers’ cognitive processes and interferes with their ability to process immediate events, thereby reducing their job performance. High job performance requires sustained effort over extended periods, requiring employees to mobilize cognitive resources such as high levels of attention and focus. This means that any kind of cognitive interference may divert employees’ attention from their current job task, resulting in lower task performance. At the same time, cognitive interference also exhausts a number of personal resources, including time, energy, and effort (Beal et al., 2005). This causes employees to lack enough personal resources to engage in additional behaviors that are beneficial to the organization, leading to a negative impact on contextual performance. Thus, we propose:

***Hypothesis 1a*** *: Leader workplace anxiety has a negative indirect effect on followers’ (a) task performance and (b) contextual performance via follower cognitive interference. Specifically, leader workplace anxiety is positively related to follower cognitive interference, which is negatively related to task performance and contextual performance.*

### The emotional reactions pathway—Emotional exhaustion

Emotion expression can also affect the behavior patterns of observers by triggering their emotional reactions, and then exerting interpersonal influence in the organization (Van Kleef, 2009; Van Kleef and Côté, 2018). When leaders display anxiety in the workplace, employees (often unconsciously and automatically) “catch” this anxiety and start to feel it as well. This process is known as emotional contagion (Van Kleef and Côté, 2018). When negative emotions spread from leaders to their followers, followers may experience a depletion of personal resources such as energy and concentration, which can lead to emotional exhaustion (Maslach et al., 2001). Furthermore, a high level of emotional exhaustion may result in employees not having enough resources to cope with work tasks, which will lead to a decline in task performance (Cropanzano et al., 2003; Halbesleben and Bowler, 2007). Besides task performance, leader workplace anxiety can also affect follower contextual performance through emotional exhaustion. Compared with task performance, contextual performance is usually outside the scope of one’s job and is not part of the formal reward system. Van Scotter et al. (2000) suggested that when and how a person engages in contextual performance is a matter of individual discretion. Therefore, when individuals experience emotional exhaustion, they lack the motivation and resources to go “above and beyond” the scope of their job. This leads us to predict:

***Hypothesis 1b*** *: Leader workplace anxiety has a negative indirect effect on follower (a) task performance and (b) contextual performance via follower emotional exhaustion. Leader workplace anxiety is positively related to follower emotional exhaustion, which is negatively related to task performance and contextual performance.*

### The moderating role of follower epistemic motivation

Although the inferential processes and emotional reactions described above can lead to lower employee performance, it is important to determine when each process is likely to produce negative work outcomes. According to the EASI framework, the interpersonal effects of emotional expression in the work environment depend on the observer’s motivation and ability to process the information conveyed by these emotions, also known as epistemic motivation (Van Kleef, 2009). Epistemic motivation influences how observers process and respond to the emotions expressed by others (Kruglanski, 1989; Van Kleef et al., 2004).

Specifically, individuals with high epistemic motivation were more likely to view their immediate emotional responses to expressed emotion as inaccurate or irrelevant (Livi et al., 2015). Instead, they are more likely to make deliberate cognitive evaluations about why the other party expressed emotion (Albarracín and Kumkale, 2003). For example, Van Kleef et al. (2009) found that when faced with leaders’ negative emotional expressions, followers with high epistemic motivation were more affected by cognitive reasoning, while followers with low epistemic motivation were more affected by emotional reactions. Van Kleef et al. (2009) also studied the influence of emotional expression on creativity. In their experiment, one participant played the role of an idea generator and the other played the role of evaluator. After the participants had an idea, the evaluators provided negative feedback to the idea producers. But they found that participants with high epistemic motivation were more engaged in the task and had more ideas after their partners expressed anger at their performance. In contrast, participants with low epistemic motivation were less involved in tasks and had fewer ideas after their partners expressed anger.

Given these findings, when followers experience cognitive interference caused by their leader’s expression of anxiety, we expect that the effect of such cognitive interference on task performance depends on each follower’s level of epistemic motivation. Employees with high epistemic motivation will be more likely to reflect on other people’s emotions and engage in deeper analysis and information processing of the root causes of leaders’ anxiety (Kruglanski and Webster, 1996). This will mitigate the negative impact of cognitive interference on task performance. In contrast, employees with low epistemic motivation are not able to analyze and process information about the reasons their leader appears anxious. These employees may continually wonder what is wrong with their leader, which will interfere with the timely completion of their job tasks. Thus, we hypothesize:

***Hypothesis 2a*** *: Follower epistemic motivation will moderate the negative relationship between cognitive interference and task performance, such that this negative relationship is weaker when epistemic motivation is higher rather than lower.*

We also expect that epistemic motivation will change the relationship between the emotional exhaustion felt by an employee and her contextual performance. The EASI theory posits that the interpersonal effects of emotional expressions depend on the observer’s ability to process and interpret the information conveyed by these expressions. The shallower the epistemic motivation, the stronger emotional reaction an observer will have to others’ displays of emotion (Hillebrandt and Barclay, 2017; Deng et al., 2020).

Emotional exhaustion depletes an individual’s resources such as energy, optimism, and self-efficacy (Halbesleben and Bowler, 2007). To preserve resources, individuals will attempt to adjust their behavior to minimize resource losses (Hobfoll, 1989; Chen et al., 2020). When employees are emotionally exhausted, they often respond by ignoring all unnecessary tasks and doing the bare minimum. These employees don’t feel they have the time or energy to engage in voluntary or proactive work behaviors which are not strictly required for them to complete their core job tasks. Therefore, employees with low epistemic motivation will be more influenced by emotional reactions to their leader’s anxiety, which will further strengthen the negative impact of emotional exhaustion on contextual performance.

***Hypothesis 2b*** *: Follower epistemic motivation will moderate the negative relationship between emotional exhaustion and contextual performance, such that this negative relationship is weaker when epistemic motivation is higher rather than lower.*

### Integrative models of the effect of leader anxiety on follower performance

In the above sections, we have proposed that employees’ epistemic motivation serves as a moderator variable affecting two mediation processes (cognitive interference and emotional exhaustion) that link leader anxiety to subordinate job performance. Our model predicts that in leader-follower interactions, workplace anxiety displayed by leaders can reduce contextual performance and task performance through emotional responses and cognitive processes. We predict that followers’ epistemic motivation levels are a boundary condition on this indirect effect. Employees with high epistemic motivation rely less on emotional states and instead process information more deliberately and systematically to guide their behavior. When they experience cognitive interference due to the leader’s expression of workplace anxiety, they will interpret this information as a signal that leaders are dissatisfied with their work results and will work harder to improve task performance.

In contrast, employees with low epistemic motivation tend to process information in a fast, relaxed, and heuristic manner. They respond to displays of emotion with their own emotions. Therefore, when they experience emotional exhaustion due to the leader’s expression of workplace anxiety, individuals with low epistemic motivation further amplify the panic and stress conveyed by their leader. This emotional exhaustion causes employees to be self-critical (Sarason, 1984) and depletes their resources which are necessary for engaging in the proactive, prosocial, and voluntary behaviors which make up an employee’s contextual performance. Therefore, we propose the following hypotheses:

***Hypothesis 3a*** *: Follower epistemic motivation will moderate the indirect effect of leader workplace anxiety on follower task performance via follower cognitive interference, such that this indirect effect will be weaker when epistemic motivation is high than when it is low.*

***Hypothesis 3b*** *: Follower epistemic motivation will moderate the indirect effect of leader workplace anxiety on follower contextual performance via follower emotional exhaustion, such that this indirect effect will be weaker when epistemic motivation is high than when it is low.*

## Materials and methods

### Sample and procedure

We collected matched supervisor-subordinate dyadic data at two time points from several high-tech enterprises located in northern China. The authors contacted each company’s human resource (HR) director and assisted the HR department with organizing the participants. Each company’s HR department helped us randomly invite dyads with direct supervisor-subordinate relationships to participate in our research. Participants were informed that we were interested in their true feelings and behaviors at work to diagnose current issues within the company and that they could quit at any time. In total, we successfully solicited 279 supervisor-subordinate dyads to participate.

Before participants filled out the questionnaire, we assured them that their answers would be kept confidential and used only for research purposes. Surveys were coded before distribution and were distributed to project team leaders and members. We collected time-lagged data at two different points to alleviate common method bias.

At time 1, we asked 279 team leaders to rate their workplace anxiety and subordinates to rate their emotional exhaustion and cognitive interference. In addition, we asked supervisors and subordinates to report their demographic information including gender, age, education, and supervisor-subordinate tenure. In total, we received 256 pairs of supervisor-subordinate dyadic data at the first time point, giving a response rate of 92%.

At Time 2 (1 month later), the 256 subordinate members who participated in the Time 1 survey assessed their task performance, contextual performance and epistemic motivation. This time, we received a total of 234 responses, for a response rate of 91%. After matching the superior-subordinate data we obtained a final sample of 228 supervisor-subordinate pairs. In the final sample, 128 subordinates (56%) were male. Their average age was 32.46 years old (*SD* = 8.35), and 156 subordinates (68%) held an undergraduate or graduate university degree. The average supervisor-subordinate tenure was 1.81 years (*SD* = 1.60). Following the procedures recommended by Rogelberg and Stanton (2007), the results of attrition analysis showed that the demographic information of subordinates who participated in both surveys was not significantly different from that of those who did not complete both surveys. Thus, there was no non-response bias in our sample.

### Measures

We followed Brislin’s (1990) procedures to translate items from English to Chinese. Unless noted, we used a 7-point Likert-type scale (1 = *strongly disagree* to 7 = *strongly agree*).

#### Workplace anxiety

We adapted the eight-item scale from Mccarthy and Goffin (2004) to measure workplace anxiety. A sample item is: “I worry about whether others consider me to be a good employee for the job.” (**α** = 0.933).

#### Emotional exhaustion

Using an eight-item scale from Maslach and Jackson (1981), we asked participants to evaluate the degree of emotional exhaustion they experienced. An example item was “I feel fatigued when I get up in the morning and have to face another day on the job” (**α** = 0.93).

#### Cognitive interference

Cognitive interference was assessed with the five-item subscale of the Cognitive Interference Questionnaire Items developed by Sarason et al. (1986b). A sample item is: “When I feel that my colleagues were nervous and anxious, I thought about how poorly I was doing” (**α** = 0.89).

#### Epistemic motivation

We employed the eleven-item scale developed by Neuberg and Newsom (1993) to measure epistemic motivation. The translated version of the scale that was used in the present study has been validated in prior work (Van Kleef et al., 2009). Examples of scale items are, “It upsets me to go into a situation without knowing what I can expect from it” (**α** = 0.82).

#### Contextual performance

We utilized the fourteen-item scale developed by Borman and Motowidlo (1997) to measure contextual performance. Examples of scale items are, “I often work with team members” and “I volunteered to help other colleagues finish their work” (**α** = 0.97).

#### Task performance

Task performance was assessed with the ten-item scale developed by Borman and Motowidlo (1997). A sample item is: “I am qualified for the assigned task” (**α** = 0.86).

#### Control variables

We controlled for the gender of the participants because previous studies have found that men and women may experience different levels of workplace anxiety for several reasons. First, biological factors such as genetic predisposition and hormonal influences may predispose women to experience higher levels of anxiety in different workplace contexts (Brooks and Schweitzer, 2011; Feeney et al., 2015). Second, women are more vulnerable to discrimination in the workplace, such as wage gaps and glass ceilings (Padavic and Reskin, 2002), which usually leads to higher levels of anxiety (Klonoff et al., 2000). Third, women often face unfair work-family balance requirements, such as being expected to undertake a lot of housework while being asked to finish their work efficiently (Baruch et al., 1987).

### Confirmatory factor analyses

To investigate the discriminant validity of focal variables, we performed a series of confirmatory factor analyses with all items as indicators. Fit indices for a six-factor model including workplace anxiety, emotional exhaustion, cognitive interference, epistemic motivation, task performance, and contextual performance were satisfactory (Hu and Bentler, 1999): χ^2^ = 1939.82, *p* < 0.01, Comparative Fit Index = 0.91, Root Mean Square Error of Approximation = 0.05, Standardized Root Mean Square Residual = 0.07. Alternative models that combined cognitive interference and emotional exhaustion, as well as contextual performance and task performance, did not improve the fit compared to the six-factor model. Therefore, the six-factor model was retained for hypothesis testing.

## Results

### Descriptive analyses

Table 1 displays means, standard deviations, correlations, and reliability estimates for study variables. Leader workplace anxiety was significantly and positively correlated with follower cognitive inference (*r* = 0.240, *p* < 0.01) and emotional exhaustion (*r* = 0.383, *p* < 0.01). Follower cognitive inference was significantly and negatively correlated with task performance and contextual performance (*r* = –0.414, *p* < 0.01 and *r* = –0.401, *p* < 0.01, respectively). Emotional exhaustion was also negatively correlated with task performance and contextual performance (*r* = –0.116, *p* = 0.081 and *r* = –0.181, *p* < 0.01, respectively).

**TABLE 1:** Means, standard deviations, correlations, and reliabilities among studied variables.

Variables	Mean	*SD*	1	2	3	4	5	6	7
Gender	1.41	0.493	–						
Leader workplace anxiety	3.33	1.57	–0.088	–					
Follower cognitive interference	3.22	1.09	–0.056	0.240**					
Follower emotional exhaustion	3.84	1.17	−0.214**	0.383**	0.295**				
Follower epistemic motivation	4.83	0.97	0.017	0.113	0.159*	0.267**			
Task performance	6.11	0.97	0.133*	−0.138*	−0.414**	–0.116	0.302**		
Contextual performance	5.69	0.91	0.067	−0.218**	−0.401**	−0.181**	0.296**	0.862***	

*N* = 228. SD, standard deviation. Gender, male = 1, female = 2.

*p < 0.05, **p < 0.01, ***p < 0.001.

### Hypothesis testing

We first adopted hierarchical regression and Hayes’s (2017) SPSS macro to test moderated mediation. By using bias-corrected 95% confidence intervals (CIs) with 5,000 bootstrap samples, our study examined the proposed indirect and conditional indirect effects.

#### Tests of mediation effects

Hypothesis 1a proposes that leader workplace anxiety indirectly affects follower task and contextual performance *via* cognitive interference. As shown in Table 2, in the parallel mediation model, leader workplace anxiety was positively related to follower cognitive interference (*b* = 0.159, *p* < 0.001), which was negatively related to task performance (*b* = –0.546, *p* < 0.001) and contextual performance (*b* = –0.065, *p* = 0.08). As reflected in Table 3, the bootstrap analyses revealed that the indirect effect of leader workplace anxiety on follower task performance through cognitive interference was significant, *b* = –0.064, 95% CI [–0.107, –0.029]. However, the indirect effect of leader workplace anxiety on contextual performance through cognitive interference was not significant, *b* = –0.004, 95% CI [–0.021, 0.036]. Thus, H1a was only supported for task performance.

**TABLE 2:** Results of hierarchical regression analyses pertaining to hypotheses 1 and 2.

Variables	Follower cognitive interference		Follower emotional exhaustion		Task performance		Contextual performance	
	*b*	*s.e.*	*b*	*s.e.*	*b*	*s.e.*	*b*	*s.e.*
***Control variables***								
Gender	–0.269***	0.07	–0.039	0.060	0.394***	0.062	0.338	0.064
***Independent variables***								
Leader workplace anxiety (LWA)	0.159***	0.049	0.244***	0.055	–0.059*	0.029	–0.103**	0.033
***Moderators***								
Follower epistemic motivation (FEM)					–0.155	0.111	0.177	0.241
***Interactions***								
Follower cognitive interference × FEM					0.104**	0.029		
Follower emotional exhaustion × FEM							0.009	0.029
***Mediators***								
Follower cognitive interference					–0.546***	0.155	–0.065	0.042
Follower emotional exhaustion					–0.237	0.046	–0.059*	0.159
*R* ^2^	0.147**		0.176**		0.687***		0.379***	

*p < 0.05, **p < 0.01, ***p < 0.001.

**TABLE 3:** Indirect and conditional indirect effects.

Indirect effect	Follower epistemic motivation	Path coefficient	95% CI
Leader workplace anxiety—Follower cognitive interference– Task performance		–0.064*	[–0.107, –0.029]
	Low	–0.055*	[–0.093, –0.021]
	High	–0.023*	[–0.048, –0.006]
	Difference	0.032*	[0.012, 0.068]
Leader workplace anxiety—Follower emotional exhaustion – Contextual performance		–0.056*	[–0.100, –0.025]
	Low	–0.027	[–0.069, 0.001]
	High	–0.022	[–0.051, –0.002]
	Difference	0.004	[–0.023, 0.031]
Leader workplace anxiety—Follower cognitive interference– Contextual performance		–0.004	[–0.021, 0.036]
			
Leader workplace anxiety—Follower emotional exhaustion– Task performance		–0.007	[–0.034, 0.021]

*n* = 228. 95% CI, bias-corrected 95% confidence interval with 1,000 resamples. *p < 0.05.

Hypothesis 1b predicts that leader workplace anxiety indirectly affects follower task and contextual performance *via* emotional exhaustion. In the parallel mediation model, leader workplace anxiety was positively related to follower emotional exhaustion (*b* = 0.244, *p* < 0.001), which was negatively related to follower task performance (*b* = –0.237, *p* < 0.01) and contextual performance (*b* = –0.059, *p* < 0.05). Similarly, the bootstrap analysis showed that the indirect effect of workplace anxiety on follower contextual performance through emotional exhaustion was significant, *b* = –0.056, 95% CI [–0.100, –0.025]. However, the specific indirect effect of leader workplace anxiety on follower task performance through emotional exhaustion was not significant, *b* = –0.007, 95% CI [–0.034, 0.021]. Thus, H1b was only supported for contextual performance.

#### Tests of moderation effects

**FIGURE 2:**
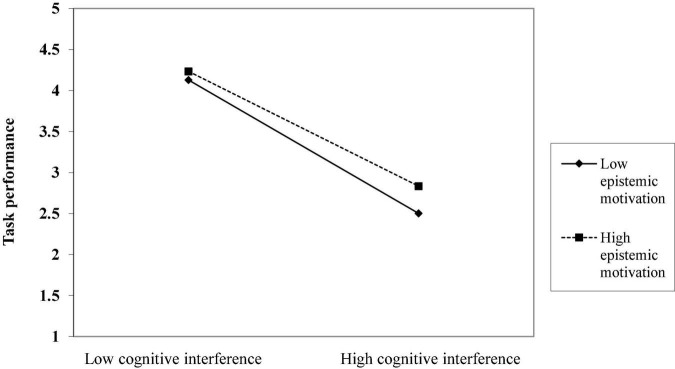
The moderating effect of epistemic motivation on the relationship between follower cognitive interference and task performance.

Hypotheses 2a predicts that follower epistemic motivation will moderate the relationship between cognitive interference and task performance, such that this negative relationship is weaker when follower epistemic motivation is higher rather than lower. Results in Table 2 indicated that the interaction term of followers’ epistemic motivation and cognitive interference on task performance was significant (*b* = 0.104, *s.e.* = 0.029, *p* < 0.01). Simple slope tests and the interaction plot depicted in Figure 2 show that when epistemic motivation was high (1 SD above the mean), cognitive interference was negatively related to employee task performance (*simple slope* = –0.642, *p* < 0.001). When epistemic motivation was low (1 *SD* below the mean), the negative relationship between followers’ cognitive interference and task performance was stronger (*simple slope* = –0.746, *p* < 0.001). Although cognitive interference was negatively related to task performance in the case of both high and low epistemic motivation, the relationship was stronger when epistemic motivation was low.

Hypothesis 2b proposes that epistemic motivation will moderate the relationship between follower emotional exhaustion and contextual performance, such that this negative relationship is weaker when epistemic motivation is higher rather than lower. Results presented in Table 2 show that the interaction term of epistemic motivation and emotional exhaustion on contextual performance was not significant (*b* = 0.009, *s.e.* = 0.029, *ns*). Thus, Hypothesis 2b was not supported.

#### Tests of conditional indirect effects

Hypotheses 3a and 3b propose indirect effects which are moderated by epistemic motivation. As shown in Table 3, the indirect effect of leader anxiety on follower task performance was both significant at higher [*b* = –0.023, 95% CI = (–0.048, –0.006)] and lower [*b* = –0.055, 95% CI = (–0.093, –0.021)] levels of epistemic motivation. Using a directional index of moderated mediation test (Hayes, 2015), we found that these effects were significantly different, *difference* = 0.032, 95% CI = [0.012, 0.068]. However, the indirect effect of leader anxiety on contextual performance was not significant at higher [*b* = –0.022, 95% CI = (–0.051, –0.002)] or lower [*b* = –0.027, 95% CI = (-0.069, 0.001)] levels of epistemic motivation. Thus, our results support Hypothesis 3a, but not 3b.

## Discussion

Drawing on the Emotion-as-Social-Information theory, our study investigated how leader workplace anxiety affects employee task performance and contextual performance through two pathways: emotional exhaustion and cognitive interference. We also found that employee epistemic motivation is a key boundary condition in this indirect relationship. Our findings offer implications about how leader anxiety can affect subordinate performance.

### Theoretical implications

First, we extend the job performance literature by examining the differential influence mechanisms of leader anxiety on employee performance. Previous research on the impact of anxiety on performance has mainly studied performance as a one-dimensional construct, with few studies suggesting that anxiety might have different effects on different dimensions of performance. Our research showed that anxiety affects different types of performance through different mechanisms. While cognitive interference mediated the effect of leader anxiety on task performance, emotional exhaustion mediated the effect of leader anxiety on contextual performance.

Second, we respond to calls for a fine-tuned framework to explore the connection between leader anxiety and follower behaviors (Liu et al., 2017). Previous studies related to this area mainly focused on the intrapersonal effects of discrete negative emotions, showing for instance that experiencing anger (Van Kleef et al., 2010) and disappointment (Van Kleef et al., 2006) influence individual judgments, creativity, and helping behavior (Calderwood et al., 2018). The present study shows that emotions can also exert their influence at the interpersonal level. Our research conclusion is consistent with previous research results, which further prove that emotions can exert interpersonal influences by transmitting information to others or triggering reciprocal emotions of others (Ekman, 1993; Fridlund, 1994; Van Kleef, 2014). We found that leader workplace anxiety negatively affects subordinate task performance and contextual performance through two separate mechanisms: cognitive interference and emotional exhaustion.

Third, our study extends EASI theory in several important ways. Specifically, the EASI framework provides a mechanism to explain how leader emotions affect follower behaviors and introduces the social function view of emotions into the leadership field. We apply EASI theory to an organizational setting and show how its predictions apply to the effect of managers’ emotional displays on their subordinates. Specifically, we found that leader anxiety can affect subordinates’ job performance by triggering both their inferential processes and emotional reactions. These findings help to generalize the principles of EASI theory to the ongoing leader-follower relationships that occur in actual organizations. Furthermore, we contribute to the literature on the EASI model by examining the effect of anxiety, a discrete emotion universally experienced in the workplace, on individual behavior. Previous studies using this model have examined the effects of discrete emotions such as anger (Van Kleef et al., 2010), disappointment (Van Kleef et al., 2006) and happiness (Van Kleef et al., 2004) on individual behavior. However, anxiety as a pervasive workplace problem has rarely been examined.

### Practical implications

Our findings also offer insights into managerial practice. First, employee performance is not only affected by the employee’s own anxiety, but also their leader’s anxiety. Therefore, leaders need to be aware that their emotional displays may have a considerable impact on their followers’ behaviors. More specifically, we suggest leaders should learn to adjust their anxiety in the workplace and try to avoid showing excessive anxiety in front of subordinates. To alleviate the anxiety of team leaders and reduce the impact of leader anxiety on employees’ work results, organizations should reduce the work pressure of leaders and guide them to effectively use their rest time to adjust their emotions (McCarthy et al., 2016). Second, organizations should not only pay attention to employee wellbeing but also provide leaders with mental health resources and channels for them to express their anxiety. For example, leaders could be provided with mindfulness courses or guidance on how to convey their anxiety to employees during face-to-face communications. Third, managers should be aware of their anxiety level and actively pursue personal strategies to reduce anxiety, such as learning new hobbies and getting quality sleep.

### Limitations and future research

Despite the theoretical contributions and practical implications discussed above, our research is not without limitations, which provide avenues for future work. First, all variables we measured were evaluated by a survey, which may lead to a certain degree of common method variance (Podsakoff et al., 2012). To alleviate this concern, we collected multi-source and multi-time data to test our hypotheses (Podsakoff et al., 2012). Nevertheless, we suggest that future research can further avoid this type of common-method bias by utilizing more objective measures to provide a more accurate assessment of the relationships between the study variables. In addition, we did not verify the causal relationship between variables, so we recommend that future researchers manipulate rather than just measure leaders’ workplace anxiety by conducting experimental studies. They may also adopt an empirical sampling method to dynamically track the impact of leaders’ intra-day mood changes on employee performance.

Second, our study only explored how subordinates’ epistemic motivations act as boundary conditions affecting the relationship between leader workplace anxiety and subordinate job performance. Therefore, future research could continue to explore other possible contextual factors, such as leaders’ emotion regulation strategies, employees’ personal traits, the quality of leader-member exchanges, and organizational climate. For example, subordinates who are more professionally adaptable and resilient are likely to be less susceptible to the severe consequences of leader workplace anxiety, and leaders who are adept at employing emotion regulation strategies can reduce the impact of their anxiety on their employees.

Third, considering the widespread use of work groups in organizations, another important direction for future research is to consider the anxiety levels of groups. In work teams, individual employees may transmit their anxiety to other team members through emotional contagion (Hatfield et al., 1992).

Fourth, we were only able to collect empirical data from China, a culture that features high power distance and a highly collectivistic orientation. Although the theoretical arguments discussed in our study are not culturally bound, we encourage future research to use cross-cultural data to demonstrate the generalizability of our findings.

## Conclusion

Today, workplace anxiety is more prominent than ever before, leading to noticeable consequences for employees and organizations. This study established, tested, and discovered that leader workplace anxiety affects subordinate performance through both emotional and cognitive mechanisms. Meanwhile, our study also contends that employee epistemic motivation acts as an antidote to the influence of leader anxiety on subordinate job performance. Overall, these findings provide further insights for researchers and practitioners to understand the consequences of workplace anxiety.

## Data availability statement

The datasets generated for this study are available on request to the corresponding author.

## Ethics statement

Ethical review and approval was not required for the study on human participants in accordance with the local legislation and institutional requirements. Written informed consent from the patients/participants or patients/participants legal guardian/next of kin was not required to participate in this study in accordance with the national legislation and the institutional requirements.

## Author contributions

SZ contributed to the data curation, formal analysis, and original draft and revision of the manuscript. LC contributed to the conceptualization and revision of the manuscript. LZ contributed to the supervision and guidelines. AS contributed to the review and editing of the manuscript. All authors contributed to the article and approved the submitted version.

## References

[B1] Albarracín D. Kumkale G. T. (2003). Affect as information in persuasion, A model of affect identification and discounting. *J. Personal. Soc. Psychol.* 84 453–469. 10.1037//0022-3514.84.3.453 12635909 PMC4797933

[B2] Amit A. Sagiv L. (2013). The role of epistemic motivation in individuals’ response to decision complexity. *Organ. Behav. Hum. Decis. Process.* 121 104–117.

[B3] Baruch G. K. Biener L. Barnett R. C. (1987). Women and gender in research on work and family stress. *Am. Psychol.* 42 130–136. 3578991 10.1037//0003-066x.42.2.130

[B4] Beal D. J. Weiss H. M. Barros E. MacDermid S. M. (2005). An episodic process model of affective influences on performance. *J. Appl. Psychol.* 90 1054–1068. 16316265 10.1037/0021-9010.90.6.1054

[B5] Borman W. C. Motowidlo S. J. (1997). Task performance and contextual performance, The meaning for personnel selection research. *Hum. Perform.* 10 99–109.

[B6] Boyd N. G. Lewin J. E. Sager J. K. (2009). A model of stress and coping and their influence on individual and organizational outcomes. *J. Vocat. Behav.* 75 197–211.

[B7] Brislin R. W. (1990). *Applied Cross-Cultural Psychology*, Vol. 14. New York, NY: Sage Publications.

[B8] Brooks A. W. Schweitzer M. E. (2011). Can Nervous Nelly negotiate? How anxiety causes negotiators to make low first offers, exit early, and earn less profit. *Organ. Behav. Hum. Decis. Process.* 115 43–54. 10.1016/j.obhdp.2011.01.008

[B9] Calderwood C. Bennett A. A. Gabriel A. S. Trougakos J. P. Dahling J. J. (2018). Too anxious to help? Off-job affective rumination is a linking mechanism between work anxiety and helping. *J. Occup.Organ. Psychol.* 91 681–687. 10.1111/joop.12220

[B10] Chen H. Richard O. C. Boncoeur O. D. Ford D. L. Jr. (2020). Work engagement, emotional exhaustion, and counterproductive work behavior. *J. Bus. Res.* 114 30–41. 10.3390/ijerph18126392 34204845 PMC8296211

[B11] Cheng B. H. McCarthy J. M. (2018). Understanding the dark and bright sides of anxiety, a theory of workplace anxiety. *J. Appl. Psychol.* 103 537–560. 10.1037/apl0000266 29355338

[B12] Cisler J. M. Olatunji B. O. Feldner M. T. Forsyth J. P. (2010). Emotion regulation and the anxiety disorders, An integrative review. *J. Psychopathol. Behav. Assess.* 32 68–82. 10.1007/s10862-009-9161-1 20622981 PMC2901125

[B13] Cropanzano R. Rupp D. E. Byrne Z. S. (2003). The relationship of emotional exhaustion to work attitudes, job performance, and organizational citizenship behaviors. *J. Appl. Psychol.* 88 160–169. 10.1037/0021-9010.88.1.160 12675403

[B14] Deng H. Walter F. Guan Y. (2020). Supervisor-directed emotional labor as upward influence: An emotions-as-social-information perspective. *J. Organ. Behav.* 41 384–402. 10.1002/job.2424

[B15] Ekman P. (1993). Facial expression and emotion. *Am. Psychol.* 48 384–392. 10.1037/0003-066X.48.4.384 8512154

[B16] Eysenck M. W. Derakshan N. Santos R. Calvo M. G. (2007). Anxiety and cognitive performance, attentional control theory. *Emotion* 7 336–353. 10.1037/1528-3542.7.2.336 17516812

[B17] Feeney J. R. McCarthy J. M. Goffin R. (2015). Applicant anxiety, Examining the sex-linked anxiety coping theory in job interview contexts. *Int. J. Select. Assess.* 23 295–305. 10.1111/ijsa.12115

[B18] Ford M. T. Cerasoli C. P. Higgins J. A. Decesare A. L. (2011). Relationships between psychological, physical, and behavioural health and work performance, A review and meta-analysis. *Work Stress* 25 185–204. 10.1080/02678373.2011.609035 28157275

[B19] Fridlund A. J. (1994). *Human Facial Expression, An Evolutionary View.* San Diego: Academic Press.

[B20] Gaddis B. Connelly S. Mumford M. D. (2004). Failure feedback as an affective event: Influences of leader affect on subordinate attitudes and performance. *Leadersh. Quart.* 15 663–686. 10.1016/j.leaqua.2004.05.011

[B21] George J. M. (2000). Emotions and leadership, The role of emotional intelligence. *Hum. Relations* 53 1027–1055. 10.1177/0018726700538001

[B22] Goldberg L. R. (1990). An alternative” description of personality”, the big-five factor structure. *J. Personal. Soc. Psychol.* 59 1216–1229. 10.1037//0022-3514.59.6.1216 2283588

[B23] Halbesleben J. R. B. Bowler W. M. (2007). Emotional exhaustion and job performance, The mediating role of motivation. *J. Appl. Psychol.* 92 93–106. 10.1037/0021-9010.92.1.93 17227154

[B24] Hareli S. Hess U. (2010). What emotional reactions can tell us about the nature of others, An appraisal perspective on person perception. *Cogn. Emot.* 24 128–140. 10.1080/02699930802613828

[B25] Hatfield E. Cacioppo J. Rapson R. L. (1992). “Primitive emotional contagion,” in *Emotion and Social Behavior*, Vol. 14 ed. Clark M. S. (Thousand Oaks, CA: SAGE), 151–177. 10.1017/CBO9781139174138

[B26] Hayes A. F. (2015). An index and test of linear moderated mediation. *Multivariate Behav. Res.* 50, 1–22. 10.1080/00273171.2014.962683 26609740

[B27] Hayes A. F. (2017). *Introduction to Mediation, Moderation, and Conditional Process Analysis, A Regression-Based Approach.* New York, NY: Guilford Press.

[B28] Hillebrandt A. Barclay L. J. (2017). Comparing integral and incidental emotions: Testing insights from emotions as social information theory and attribution theory. *J. Appl. Psychol.* 102 732–752. 10.1037/apl0000174 28054819

[B29] Hillebrandt A. Barclay L. J. (2022). How COVID-19 can promote workplace cheating behavior via employee anxiety and self-interest: And how prosocial messages may overcome this effect. *J. Organ. Behav.* 43 858–877. 10.1002/job.2612 35574191 PMC9088701

[B30] Hobfoll S. E. (1989). Conservation of resources, a new attempt at conceptualizing stress. *Am. Psychol.* 44 513–524. 10.1037/0003-066X.44.3.513 2648906

[B31] Hu L. T. Bentler P. M. (1999). Cutoff criteria for fit indexes in covariance structure analysis, Conventional criteria versus new alternatives. *Struct. Equ. Model.* 6 1–55. 10.1080/10705519909540118

[B32] Klonoff E. A. Landrine H. Campbell R. (2000). Sexist discrimination may account for well-known gender differences in psychiatric symptoms. *Psychol. Women Quart.* 24 93–99. 10.1111/j.1471-6402.2000.tb01025.x

[B33] Kouchaki M. Desai S. D. (2015). Anxious, threatened, and also unethical: How anxiety makes individuals feel threatened and commit unethical acts. *J. Appl. Psychol.* 100 360–375. 10.1037/a0037796 25243997

[B34] Kruglanski A. W. (1989). *Lay Epistemics and Human Knowledge, Cognitive and Motivational Bases.* New York, NY: Plenum. 10.1007/978-1-4899-0924-4

[B35] Kruglanski A. W. Webster D. M. (1996). Motivated closing of the mind,‘ Seizing’ and ‘Freezing’. *Psychol. Rev.* 103 263–283. 10.1037/0033-295x.103.2.263 8637961

[B36] Lazarus R. S. (1991). *Emotion and Adaptation.* Oxford: Oxford University Press.

[B37] Lin Y. E. Tseng C. N. Wang M. F. Wu S. F. V. Jane S. W. Chien L. Y. (2020). Anxiety and work stress among newly employed nurses during the first year of a residency program, A longitudinal study. *J. Nursing Manag.* 28 1598–1606. 10.1111/jonm.13114 32743848

[B38] Liu C. Hu C. Xie W. Liu T. He W. (2020). The Moderated-Mediation Effect of Workplace Anxiety and Regulatory Focus in the Relationship between Work-Related Identity Discrepancy and Employee Innovation. *Int. J. Environ.Res. Public Health* 17:6121. 10.3390/ijerph17176121 32842458 PMC7503295

[B39] Liu W. Song Z. Li X. Liao Z. (2017). Why and when leaders’ affective states influence employee upward voice. *Acad. Manag. J.* 60, 238–263. 10.5465/amj.2013.1082

[B40] Livi S. Kruglanski A. W. Pierro A. Mannetti L. Kenny D. A. (2015). Epistemic motivation and perpetuation of group culture: Effects of need for cognitive closure on trans-generational norm transmission. *Organ. Behav. Hum. Decis. Process.* 129 105–112. 10.1016/j.obhdp.2014.09.010

[B41] Mao J. Chang S. Gong Y. Xie J. L. (2021). Team job-related anxiety and creativity: Investigating team-level and cross-level moderated curvilinear relationships. *J. Organ. Behav.* 42 34–47. 10.1002/job.2489

[B42] Maslach C. Jackson S. E. (1981). The measurement of experienced burnout. *J. Organ. Behav.* 2 99–113. 10.1002/job.4030020205

[B43] Maslach C. Schaufeli W. B. Leiter M. P. (2001). Job burnout. *Annu. Rev. Psychol.* 52 397–422. 10.1146/annurev.psych.52.1.397 11148311

[B44] Mayseless O. Kruglanski A. W. (1987). What makes you so sure? Effects of epistemic motivations on judgmental confidence. *Organ. Behav. Hum. Decis. Process.* 39 162–183. 10.1016/0749-5978(87)90036-7

[B45] Mccarthy J. Goffin R. (2004). Measuring job interview anxiety, beyond weak knees and sweaty palms. *Personnel Psychol.* 57 607–637.

[B46] McCarthy J. M. Trougakos J. P. Cheng B. H. (2016). Are anxious workers less productive workers? It depends on the quality of social exchange. *J. Appl. Psychol.* 101 279–291. 10.1037/apl0000044 26375962

[B47] Motowidlo S. J. Borman W. C. Schmit M. J. (1997). A theory of individual differences in task and contextual performance. *Hum. Perform.* 10 71–83. 10.1207/s15327043hup1002_1

[B48] Motowidlo S. J. Packard J. S. Manning M. R. (1986). Occupational stress, its causes and consequences for job performance. *J. Appl. Psychol.* 71 618–629. 10.1037/0021-9010.71.4.618 3804934

[B49] Motowidlo S. J. Van Scotter J. R. (1994). Evidence that task performance should be distinguished from contextual performance. *J. Appl. Psychol.* 79 475–480. 10.1037/0021-9010.79.4.475 10948797

[B50] Muschalla B. Linden M. (2012). Specific job anxiety in comparison to general psychosomatic symptoms at admission, discharge and six months after psychosomatic inpatient treatment. *Psychopathology* 45 167–173. 10.1159/000330263 22398433

[B51] Neuberg S. L. Newsom J. T. (1993). Personal need for structure, individual differences in the desire for simple structure. *J. Personal. Soc. Psychol.* 65 113–131. 10.1097/WNR.0000000000000345 25730679

[B52] Newcombe M. J. Ashkanasy N. M. (2002). The role of affect and affective congruence in perceptions of leaders: An experimental study. *Leadersh. Quart.* 13 601–614. 10.1016/S1048-9843(02)00146-7

[B53] Padavic I. Reskin B. (2002). *Women and Men at Work*, 2nd Edn. Thousand Oaks, CA: Pine Forge Press. 10.4135/9781452233857

[B54] Podsakoff P. M. MacKenzie S. B. Podsakoff N. P. (2012). Sources of method bias in social science research and recommendations on how to control it. *Annu. Rev. Psychol.* 63 539–569. 10.1146/annurev-psych-120710-100452 21838546

[B55] Rodell J. B. Judge T. A. (2009). Can “good” stressors spark “bad” behaviors? The mediating role of emotions in links of challenge and hindrance stressors with citizenship and counterproductive behaviors. *J. Appl. Psychol.* 94 1438–1451. 10.1037/a0016752 19916654

[B56] Rogelberg S. G. Stanton J. M. (2007). Introduction, understanding and dealing with organizational survey nonresponse. *Organ. Res. Methods* 10 195–209. 10.1177/1094428106294693

[B57] Sarason I. G. (1984). Stress, anxiety, and cognitive interference: Reactions to tests. *J. Pers. Soc. Psychol.* 46, 929–938. 10.1037//0022-3514.46.4.929 6737201

[B58] Sarason I. G. Pierce G. R. Sarason B. R. (2014). *Cognitive Interference, Theories, Methods, and Findings.* Oxfordshire: Routledge. 10.4324/9781315827445

[B59] Sarason I. G. Potter E. H. Sarason B. R. (1986a). Recording and recall of personal events, Effects on cognitions and behavior. *J. Personal. Soc. Psychol.* 51 347–356. 10.1037/0022-3514.51.2.347

[B60] Sarason I. G. Sarason B. R. Keefe D. E. Hayes B. E. Shearin E. N. (1986b). Cognitive interference, situational determinants and traitlike characteristics. *J. Personal. Soc. Psychol.* 51 215–226. 10.1037/0022-3514.51.1.215

[B61] Sarason I. G. Sarason B. R. Pierce G. R. (1990). Anxiety, cognitive interference, and performance. *J. Soc. Behav. Personal.* 5 1–13.

[B62] Sarason I. G. Sarason B. R. Pierce G. R. (1995). “Cognitive Interference,” in *International Handbook of Personality and Intelligence*, eds Saklofske D. H. Zeidner M. (Boston, MA: Springer), 285–296. 10.1007/978-1-4757-5571-8_14

[B63] Scholten L. Van Knippenberg D. Nijstad B. A. De Dreu C. K. (2007). Motivated information processing and group decision-making, Effects of process accountability on information processing and decision quality. *J. Exp. Soc. Psychol.* 43 539–552. 10.1186/s12913-016-1423-5 27409075 PMC4943498

[B64] Schwarzmüller T. Brosi P. Welpe I. M. (2018). Sparking anger and anxiety: Why intense leader anger displays trigger both more deviance and higher work effort in followers. *J. Bus. Psychol.* 33 761–777.

[B65] Spielberger C. D. (1966). *Theory and Research on Anxiety. Anxiety and Behavior.* New York, NY: Academic press.

[B66] Spielberger C. D. (1985). “Anxiety, cognition and affect: A state-trait perspective,” in *Anxiety and the Anxiety Disorders*, eds Tuma A. H. Maser J. (Hillsdale, NJ: Lawrence Erlbaum), 171–182.

[B67] Spielberger C. D. (2013). *Anxiety, Current trends in Theory and Research.* Amsterdam, NL: Elsevier.

[B68] Turner J. R. Müller R. (2005). The project manager’s leadership style as a success factor on projects, A literature review. *Project Manag. J.* 36 49–61.

[B69] Van Kleef G. A. (2009). How emotions regulate social life, The emotions as social information (EASI) model. *Curr. Direct. Psychol. Sci.* 18 184–188.

[B70] Van Kleef G. A. (2014). Understanding the positive and negative effects of emotional expressions in organizations, EASI does it. *Hum. Relations* 67 1145–1164.

[B71] Van Kleef G. A. Anastasopoulou C. Nijstad B. A. (2010). Can expressions of anger enhance creativity? A test of the emotions as social information (EASI) model. *J. Exp. Soc. Psychol.* 46 1042–1048.

[B72] Van Kleef G. A. Côté S. (2018). Emotional dynamics in conflict and negotiation, Individual, dyadic, and group processes. *Annu. Rev. Organ. Psychol. Organ. Behav.* 5 437–464. 10.1016/j.adolescence.2008.08.005 18951625

[B73] Van Kleef G. A. De Dreu C. K. Manstead A. S. (2004). The interpersonal effects of anger and happiness in negotiations. *J. Personal. Soc. Psychol.* 86 57–76. 10.1037/0022-3514.86.1.57 14717628

[B74] Van Kleef G. A. De Dreu C. K. Manstead A. S. (2006). Supplication and appeasement in conflict and negotiation, The interpersonal effects of disappointment, worry, guilt, and regret. *J. Personal. Soc. Psychol.* 91 124–142. 10.1037/0022-3514.91.1.124 16834484

[B75] Van Kleef G. A. Homan A. C. Beersma B. Van Knippenberg D. Van Knippenberg B. Damen F. (2009). Searing sentiment or cold calculation? The effects of leader emotional displays on team performance depend on follower epistemic motivation. *Acad. Manag. J.* 52 562–580.

[B76] Van Scotter J. Motowidlo S. J. Cross T. C. (2000). Effects of task performance and contextual performance on systemic rewards. *J. Appl. Psychol.* 85 526–535. 10948797 10.1037/0021-9010.85.4.526

[B77] Wang L. Lin H. Jiang W. (2021). Effects of project leader workplace anxiety on project team member organizational citizenship behavior, A moderated mediation model. *Project Manag. J.* 52 340–353.

[B78] Wang Z. Singh S. N. Li Y. J. Mishra S. Ambrose M. Biernat M. (2017). Effects of employees’ positive affective displays on customer loyalty intentions, An emotions-as-social-information perspective. *Acad. Manag. J.* 60 109–129.

